# The relationship between the epigenetic aging biomarker “grimage” and lung function in both the airway and blood of people living with HIV: An observational cohort study

**DOI:** 10.1016/j.ebiom.2022.104206

**Published:** 2022-08-06

**Authors:** Ana I Hernández Cordero, Chen Xi Yang, Julia Yang, Xuan Li, Steve Horvath, Tawimas Shaipanich, Julia MacIsaac, David Lin, Lisa McEwen, Michael S. Kobor, Silvia Guillemi, Marianne Harris, Wan Lam, Stephen Lam, Ma'en Obeidat, Richard M. Novak, Fleur Hudson, Hartwig Klinker, Nila Dharan, Julio Montaner, S.F. Paul Man, Ken Kunisaki, Don D. Sin, Janice M. Leung, INSIGHT START Pulmonary and Genomic Substudy Groups, J.V. Baker, D. Duprez, A. Carr, J. Hoy, M. Dolan, A. Telenti, C. Grady, G. Matthews, J. Rockstroh, W.H. Belloso, J.M. Kagan, E. Wright, B. Brew, R.W. Price, K. Robertson, L. Cysique, K.M. Kunisaki, J.E. Connett, D.E. Niewoehner, Endpoint Review Committee, A. Lifson, W.H. Belloso, R.T. Davey, D. Duprez, J.M. Gatell, J. Hoy, C. Pedersen, R.W. Price, R. Prineas, J. Worley

**Affiliations:** a Centre for Heart Lung Innovation, St. Paul's Hospital and University of British Columbia, Vancouver, British Columbia, Canada; b Department of Biostatistics, Fielding School of Public Health, University of California Los Angeles (UCLA), Los Angeles, CA 90095, United States; c Department of Human Genetics, David Geffen School of Medicine, University of California Los Angeles (UCLA), Los Angeles, CA 90095, United States; d Division of Respiratory Medicine, Department of Medicine, University of British Columbia, Vancouver, British Columbia, Canada; e Centre for Molecular Medicine and Therapeutics, University of British Columbia, Vancouver, British Columbia, Canada; f British Columbia Centre for Excellence in HIV/AIDS, Providence Health Care, Vancouver, British Columbia, Canada; g Faculty of Medicine, University of British Columbia, Vancouver, British Columbia, Canada; h British Columbia Cancer Research Centre and the University of British Columbia, Vancouver, British Columbia; i Section of Infectious Diseases, University of Illinois at Chicago, Chicago, IL, United States; j MRC Clinical Trials Unit, University College London, London, UK; k Medizinische Klinik und Poliklinik II, Universitatsklinikum Wurzburg, Wurzburg, Germany; l The Kirby Institute, UNSW Sydney, Sydney, New South Wales, Australia; m Minneapolis Veterans Affairs Health Care System, Section of Pulmonary, Critical Care and Sleep Medicine and the Division of Pulmonary, Allergy, Critical Care and Sleep Medicine, Department of Medicine, University of Minnesota, Minneapolis, MN, United States

**Keywords:** HIV, COPD, Epigenetic age, Blood, Airway

## Abstract

**Background:**

Age-related comorbidities such as chronic obstructive pulmonary disease (COPD) are common in people living with human immunodeficiency virus (PLWH). We investigated the relationship between COPD and the epigenetic age of the airway epithelium and peripheral blood of PLWH.

**Methods:**

Airway epithelial brushings from 34 PLWH enrolled in the St. Paul's Hospital HIV Bronchoscopy cohort and peripheral blood from 378 PLWH enrolled in The Strategic Timing of Antiretroviral Treatment (START) study were profiled for DNA methylation. The DNA methylation biomarker of age and healthspan, GrimAge, was calculated in both tissue compartments. We tested the association of GrimAge with COPD in the airway epithelium and airflow obstruction as defined by an FEV_1_/FVC<0.70, and FEV_1_ decline over 6 years in blood.

**Findings:**

The airway epithelium of PLWH with COPD was associated with greater GrimAge residuals compared to PLWH without COPD (Beta=3.18, 95%CI=1.06-5.31, *P*=0.005). In blood, FEV_1_/FVC<LLN was associated with greater GrimAge residuals (Beta=1.74, 95%CI=0.37-3.24, *P*=0.019). FEV_1_ decline was inversely correlated with GrimAge residuals in blood (r=−0.13, *P*=0.012). PLWH who had normal lung function but who subsequently developed an FEV_1_/FVC<0.70 over the course of 6 years had higher GrimAge residuals at baseline (Beta=2.33, 95%CI=0.23-4.44, *P*=0.031).

**Interpretation:**

GrimAge may reflect lung and systemic epigenetic changes that occur with advanced airflow obstruction and may help to identify PLWH with a higher risk of developing COPD.

**Funding:**

Canadian Institutes of Health Research and the British Columbia Lung Association. The START substudy was funded by NIH grants: UM1-AI068641, UM1-AI120197, and RO1HL096453.

**Research in context**

**Evidence before this study**

People living with human immunodeficiency virus (PLWH) have an increased risk of developing age-related conditions such as chronic obstructive pulmonary disease (COPD) compared to the general population. This has given rise to the theory that HIV itself is associated with an accelerated aging phenomenon. Epigenetic clocks based on DNA methylation have been previously used to study accelerated aging in both HIV and COPD. In COPD specifically, higher epigenetic age has been associated with mortality. A systematic search of HIV-associated COPD and epigenetic aging identified only two manuscripts which showed that 1) inflammatory genes are at least in part associated with epigenetic age acceleration and 2) that the airway epithelium in HIV-associated COPD is characterized by accelerated ageing. However, whether epigenetic clocks can serve as a global marker of lung injury identifying PLWH with lung disease in both airway and blood compartments remains unknown.

**Added value of this study**

In two separate cohorts of PLWH, we found that an epigenetic clock and biomarker of mortality, GrimAge, is associated with chronic obstructive pulmonary disease (COPD) in airway epithelial cells and with lung function decline in blood. GrimAge acceleration in PLWH without known lung disease was shown to be associated with the future development of airflow obstruction.

**Implications of all the available evidence**

Our findings showed that as a biomarker of lung disease, GrimAge has the ability to translate from airway to blood compartments, suggesting a global epigenetic aging phenomenon in PLWH with COPD. GrimAge may therefore help to identify PLWH at risk of developing COPD. Future validation of GrimAge performance to predict poor outcomes in PLWH with COPD would be warranted.

Alt-text: Unlabelled box

## Introduction

Due to advances in antiretroviral therapy (ART), people living with human immunodeficiency virus (PLWH) now have life expectancies close to those of the general population.^1^^,^^2^ However, age-related comorbidities such as chronic obstructive pulmonary disease (COPD) are becoming more common.^3^ COPD is characterized by persistent airflow obstruction and respiratory symptoms and in PLWH is associated with a higher risk of mortality compared to uninfected individuals.^4^ Previous research has suggested that in addition to smoking, HIV could represent an additional risk factor contributing to downstream airway injury in HIV-associated COPD.^5^ The prevalence of age-related conditions in HIV has given rise to the theory that HIV itself might also be associated with an accelerated aging phenomenon.^6^

Epigenetic changes such as the dynamic DNA methylation or demethylation of specific sites along the genome have been shown to strongly correlate with age, thus DNA methylation is a powerful tool with which to study the aging process. DNA methylation changes also represent a molecular marker by which we can link aging with the environmental and genetic factors leading to COPD.^7^^,^^8^ These properties of DNA methylation have been explored in the context of epigenetic clocks, which provide a compelling indication of biological age.^9^, ^10^, ^11^ In the past, we have used DNA methylation to show that HIV is associated with epigenetic age acceleration compared to uninfected individuals^12^^,^^13^ and that airflow obstruction is also associated with global hypomethylation in blood.^14^

Multiple epigenetic clocks based on DNA methylation have now been developed to explore accelerated aging in both HIV and COPD.^10^^,^^15^, ^16^, ^17^ An epigenetic clock called DNA methylation GrimAge (DNAmGrimAge) was specifically designed to connect the relationship between “grim” events such as death and biological age and its derivation was based on smoking pack-years and multiple inflammatory proteins associated with mortality.^16^ Previously shown to be associated with COPD,^18^ its performance has not yet been explored in airway-derived samples nor in PLWH. In this study, we hypothesized that DNAmGrimAge in the peripheral blood and airway epithelium of PLWH reflects the lung health status of this population.

## Methods

The overall observational cohort study design is shown in Supplementary Figure 1.

### Study cohorts

#### St Paul's Hospital HIV bronchoscopy study cohort

This was an observational cohort study that took place in Vancouver, Canada, between 2014 and 2018 in which airway epithelial cells were collected from PLWH with and without COPD, in addition to COPD only and negative controls.^19^ For this manuscript we used all consecutive PLWH enrolled in the main cohort (n=34) of whom 18 were identified as having COPD; no other inclusion/exclusion criteria were used. COPD was defined based on a pulmonologist's diagnosis of COPD and either a pre-bronchodilator forced expiratory volume in one second (FEV_1_)/forced vital capacity (FVC)≤lower limit of normal (LLN) or clear evidence of emphysema on computed tomography imaging on visual inspection. Airway epithelial brushings from each participant were obtained via bronchoscopy through previously published methods.^19^, ^20^, ^21^

#### The Strategic Timing of Antiretroviral Treatment (START) study cohort

This cohort consisted of all 378 adults with HIV who were enrolled in the genomic and pulmonary sub-studies of the international, multicentre START randomized controlled trial (Clinicaltrials.gov NCT00867048)^12^^,^^14^^,^^22^, ^23^, ^24^; no other inclusion/exclusion criteria were used for the downstream analyses. Briefly, the START cohort included adult PWLH with CD4 T cell counts > 500 cells/mm^3^ who had not yet been exposed to ART.^25^ Participants were enrolled between 2009-2015. All 378 participants provided whole blood samples for genomic profiling at baseline and underwent spirometry testing annually for up to 6 years.^25^ Participants were characterized as having airflow obstruction based on an FEV_1_/FVC≤LLN with additional analyses also using an FEV_1_/FVC<0.70.^26^^,^^27^ Lung function decline was defined as the slope of decline over time obtained from the regression of FEV_1_ on time.

### DNA methylation profiling

DNA was extracted from 1) the airway epithelial brushings (bronchoscopy cohort) and 2) whole blood samples obtained upon entry into the trial (START cohort) using the DNeasy Blood and Tissue Kit (Qiagen, Hilden, Germany). Unmethylated cytosine residues present in the DNA extract were converted to uracil using the EZ DNA Methylation Kit (Zymo, Irvine, California). The Illumina Infinium MethylationEPIC BeadChip microarray was used to profile 863,904 DNA methylation sites (CpG probes) from the airway epithelial brushings and whole blood samples and was performed by technicians blinded to the clinical data. Airway epithelial brushings and blood samples were processed separately. Filtering and quality control measures were performed according to previously described methods.^12^^,^^14^^,^^19^ Briefly, the beta-value for the CpG probes was calculated as the ratio of methylation probe intensity to the overall intensity ranging from 0 (fully unmethylated) to 1 (fully methylated). CpG probes were filtered based on their detection quality and probes with a detection P>1 × 10^−10^ were excluded from downstream analyses. Non-CpG, XY-linked, single nucleotide polymorphism, and cross-hybridization probes were also removed. Background correction, normalization, and batch correction were applied using the Normal-exponential out-of-band,^28^ Beta-Mixture Quantile Normalization,^29^ and ComBat^30^ methods, respectively.

### Epigenetic clock and statistical analyses

We calculated DNA methylation GrimAge (DNAmGrimAge) for each sample based on methods published by Lu *et al* by imputing the DNA methylation profiles into the Horvath's Laboratory's website (https://dnamage.genetics.ucla.edu/home; date last accessed 11 June 2022).^16^ This tool calculates DNAmGrimAge as the linear combination of the weighted average of 1,030 selected CpGs. DNAmGrimAge was developed in a two stage approach. The first stage used elastic net regression to regress 88 plasma proteins and smoking pack-years on DNA methylation, chronological age, and gender, which identified 12 DNA methylation-based biomarkers at r>0.35. In the second stage, time-to-death due to all-cause mortality was regressed on chronological age, sex, and the DNA methylation-based biomarkers of smoking pack-years and the 12 identified plasma proteins using elastic net regression. The final model selected the covariates chronological age, sex, and the DNA methylation-based biomarkers of smoking pack-years, adrenomedullin, beta-2 microglobulin, cystatin C, growth differentiation factor 15, leptin, plasminogen activator inhibitor 1, and tissue inhibitor metalloproteinase 1 as its final model.^16^ DNAmGrimAge is calibrated in years, strongly predicts healthspan and lifespan,^16^ and has been used to investigate epigenetic age in multiple diseases including COPD.^31^, ^32^, ^33^, ^34^

The output measure, GrimAge residual, was defined as the residuals from the regression of DNAmGrimAge on chronologic age, where higher values of the GrimAge residuals indicate older epigenetic age than expected based on an individual's chronological age (Supplementary Figure 2). This output measure has been previously used as a measure of epigenetic age acceleration.^16^^,^^34^ In airway epithelial cells, we investigated the relationship between GrimAge residuals and 1. COPD and 2. lung function (FEV_1_ and FEV_1_/FVC) using linear models adjusted for age, sex, and body mass index (BMI). For the tests on lung function traits, we used fully adjusted GrimAge residuals (DNAmGrimAge ∼ Age + Sex + BMI) as the response variable in a univariable model (GrimAge residuals ∼ lung function) to avoid collinearity. Participants with missing lung function (n=3, all individuals without COPD as confirmed in their medical records and by the absence of emphysema on chest computed tomographic imaging) were not used in the lung function trait analyses, but were included in the COPD analyses. Similarly, we tested the association between DNAmGrimAge in blood and airflow obstruction (FEV_1_/FEV<LLN or 0.70), lung function (FEV_1_ and FEV_1_/FVC), and FEV_1_ decline. Because DNA methylation can vary depending on cell type proportions in the blood, analyses were adjusted for estimated cell proportions (CD8T, CD4T, NK, B, Monocytes, and Granulocytes) using the first 5 principal components (PC1 to PC5) obtained from a principal component analysis.^35^ Further analyses in the bronchoscopy cohort adjusted for smoking status and inhaled corticosteroid use while in the START cohort, models were also created to adjust for smoking status. In addition, we show the extent to which DNAmGrimAge residuals and lung function traits were linearly related by providing the correlation coefficients and corresponding P-values. Significant associations were defined at *P*<0.05.

We used the receiver operating characteristic curve (ROC) to evaluate the prediction potential of GrimAge residuals (“pROC” R package). For the airway epithelial cells, we calculated the area under the curve (AUC) for COPD by first performing a logistic regression to obtain predictions based on 1) the GrimAge residuals alone, 2) chronological age, sex, BMI, and smoking status, and 3) GrimAge residuals with chronological age, sex, BMI, and smoking status (full model). In blood, we assessed GrimAge residual's prediction potential for: 1) airflow obstruction; 2) fast vs. slow FEV_1_ decline (fast decline defined as >40mL/year^36^); 3) PLWH who did not have airflow obstruction at baseline, but who developed airflow obstruction in the subsequent years of the study. The first model used GrimAge residuals alone, then a full model was performed by adding chronological age, sex, race, BMI, and smoking status. For lung function decline, the full model was also adjusted for baseline FEV_1_. Significant associations were defined at P<0.05.

In the START cohort, we further explored the relationship between HIV-associated factors (CD4 cell count, CD4 nadir, CDT4/CD8T ratio, and HIV viral load) and DNAmGrimAge by using a linear model adjusted for age, sex, BMI, and race. We used a likelihood ratio test to evaluate the association between Hepatitis C co-infection, hypertension, and DNAmGrimAge.

The description of each statistical model is presented in Supplementary Table 1.

### Power calculation

Based on our previous work estimating the Horvath DNA methylation skin & blood clock in the airway epithelium of PLWH with and without COPD,^19^ we calculated a Cohen d estimate (effect size=-1.35) and used a t-test power calculation. We determined that at least 10 samples per study group in the bronchoscopy cohort would be necessary to achieve an 80% power to detect a difference between PLWH with and without COPD, assuming an α=0.05.

### Ethics

For the St. Paul's Hospital HIV Bronchoscopy Study Cohort, participants consented to the bronchoscopic collection of research specimens under the University of British Columbia Research Ethics Board Certificates H11-02713 and H15-02166. The START trial was approved by the institutional review board of each participating site (see the Supplementary File for a full list of sites). Written informed consent was obtained from each participant in both cohorts.

### Role of funding sources

The Canadian Institutes of Health Research and the British Columbia Lung Association provided the funds relevant to the recruitment of participants and specimens and data collection that correspond to the St. Paul's Hospital HIV Bronchoscopy Study cohort. The CIHR also provided the funds to perform the methylation profiling of both the bronchoscopy and START cohorts. NIH grants UM1-AI068641, UM1-AI120197, and RO1HL096453 provided the funds for the enrolment and specimens and data collection for the START substudy. The funding sources did not contribute to the study design, data collection, data analyses, interpretation, or manuscript writing.

## Results

### Study cohorts

The bronchoscopy cohort included 34 PLWH, of whom 18 had COPD (COPD+) and 16 did not (COPD-) (Table 1). Overall, the age, proportion of females, smoking status, use of inhaled corticosteroids (ICS), and ART were similar between COPD+ and COPD-, while the FEV_1_/FVC ratio was lower in COPD+ participants. The number of participants with undetectable HIV viral loads was similar between the two groups.

**Table 1:** Study cohort demographics.

Bronchoscopy cohort (*n*=34)		
	COPD+	COPD-
N	18	16
Age (median, IQR)	56 (52-63)	57 (53-61)
Females, %	22%	13%
Smoking status	-	-
Current, %	61%	19%
Former, %	28%	50%
Never, %	6%	25%
Smoking pack-year history	38 (30-49)	4.50 (0-23)
Race (Caucasian), %	94%	100%
BMI (KG/M2)	24.06 (19.16-26.68)	26.81 (23.09-27.52)
FEV1, ML	2655 (1925-3058)	2870 (2710-3550)
FEV1, %	76.30% (58.00-91.05)	85.00% (81.00-92.00)
FVC, ML	3940 (3245-4765)	4010 (3550-4710)
FVC, %	90.95% (78.75-102.50)	87.00% (81.40-103.00)
FEV1/FVC, %	67.04% (60.38-75.53)	75.00% (71.51-80.65)
Undetectable HIV viral load, %	89%	63%
CD4 cell count (cells/MM3)	450 (260-510)	460 (110-620)
On ART, %	94%	81%
On ICS, %	22%	19%

Start cohort (*n*=378)		
	Airflow obstruction (FEV_1_/FVC<LLN)	No airflow obstruction (FEV_1_/FVC≥LLN)
N	31	347
Age (mean ± SD)	40 (34-49)	37 (31-54)
Females, %	9.68%	8.07%
Smoking status		
Current, %	61%	44%
Former, %	6%	20%
Never, %	32%	36%
Pack-year history	5 (0-23)	2 (0-9)
Race		
Black, %	19%	18%
Asian, %	0%	1%
Caucasian, %	68%	62%
Hispanic, %	10%	17%
Other, %	3%	2%
BMI (KG/M^2^)	24.11 (22.21-26.36)	24.49 (22.41-27.50)
FEV_1_, ML	3120 (2445-3795)	3830 (3350- 4330)
FEV_1_, % Predicted	81.68% (69.29-90.41)	96.64% (88.88-104.14)
FVC, ML	4910 (3740-5790)	4690 (4125-5345)
FVC, % Predicted	97.70% (86.21-108.52)	95.95% (88.10-103.59)
FEV_1_/FVC Ratio	0.6731 (0.6470- 0.6913)	0.8193 (0.7815- 0.8581)
Hepatitis C	3%	4%
Hypertension	0%	10%
CD4 cells/MM^3^	655.0 (580.5- 719.8)	637.5 (585.0-740.0)
HIV RNA viral load, copies/MM^3^	20200 (4856- 60598)	17090 (4456-56402)

ART: antiretroviral therapy BMI: body mass index. ICS: Inhaled corticosteroids. SD: standard deviation. Spirometry corresponds to pre-bronchodilator measurements. Median and interquartile range are shown for non-normally distributed variables. Race was based on participant's self-assessment.

The START cohort was stratified based on airflow obstruction by on two criteria [FEV_1_/FVC<LLN and FEV_1_/FVC<0.70 (Supplementary Table 2)]. Overall, PLWH with airflow obstruction were statistically significantly older (Table 1). CD4 cell counts and HIV viral loads were similar between the groups.

### Airway epithelial GrimAge residuals are associated with COPD in PLWH

**Figure 1:**
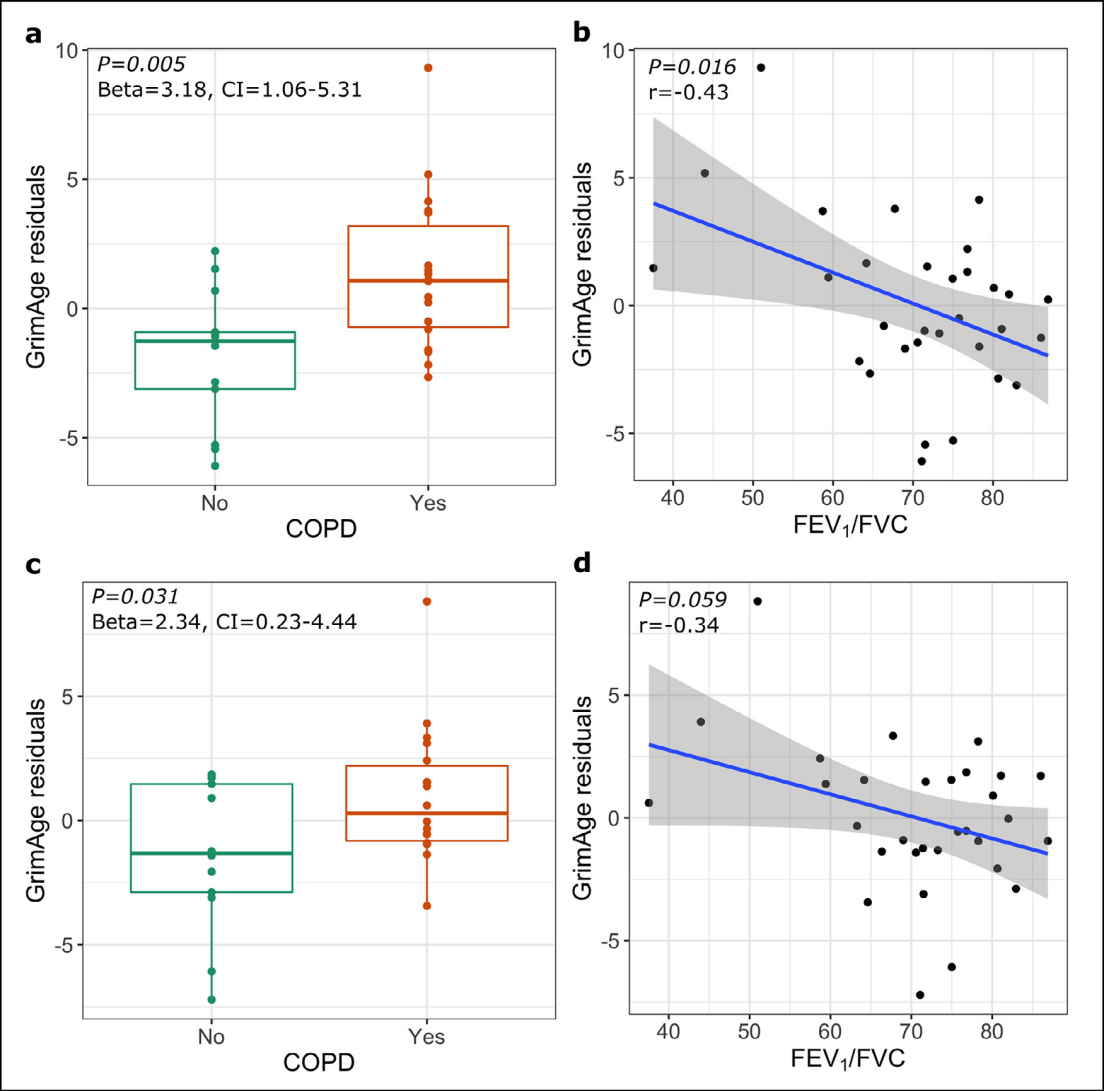
**Airway epithelium GrimAge residuals in PLWH with COPD**. (a) GrimAge residuals association with COPD (N COPD positive =18, N COPD negative = 13). (b) GrimAge residuals association with FEV_1_/FVC (*n*=31). (a) and (b) represent analyses adjusted for age, sex, and BMI. (c) and (d) represent analyses adjusted for age, sex, bmi and smoking status. R and *P*-values corresponds to the univariate linear regression p-value for the variables presented in x and y axis in (a), (b), (c) and (d).

The airway epithelial DNAmGrimAge of PLWH was highly correlated with chronological age (Supplementary Figure 3) (*r*=0.88, *P*<0.001). COPD was statistically significantly associated with higher GrimAge residuals (Beta=3.18, 95%CI=1.06-5.31, *P*=0.005,) (Figure 1a). Although FEV_1_ was not statistically significantly associated with GrimAge residuals (*r*=-0.33, *P*=0.070), decreased FEV_1_/FVC ratio was (*r*=−0.43, *P*=0.016) (Figure 1b). These results were still statistically significant when the analyses were adjusted for ICS use (Supplementary Table 3). Furthermore, after adjusting for cigarette smoking, the association between GrimAge residuals and COPD remained statistically significant (Beta=2.33, 95%CI=0.23-4.44, *P*=0.031) (Figure 1c), while the FEV_1_/FVC demonstrated a borderline statistically significant association (*r*=−0.34, *P=*0.059) (Figure 1d). The AUC for COPD based only on age, sex, BMI, and smoking status showed a prediction performance of 0.88 (95% CI=0.75-1), while the AUC for COPD based solely on GrimAge residuals was 0.77 (95% CI =0.60-0.95). The AUC improved to 0.94 (95% CI=0.86-1) when chronological age, sex, BMI, and smoking status were added to GrimAge residuals. (Supplementary Figure 4a, 4b and 4c).

### Blood DNA methylation GrimAge residuals are associated with airflow obstruction in PLWH

**Figure 2:**
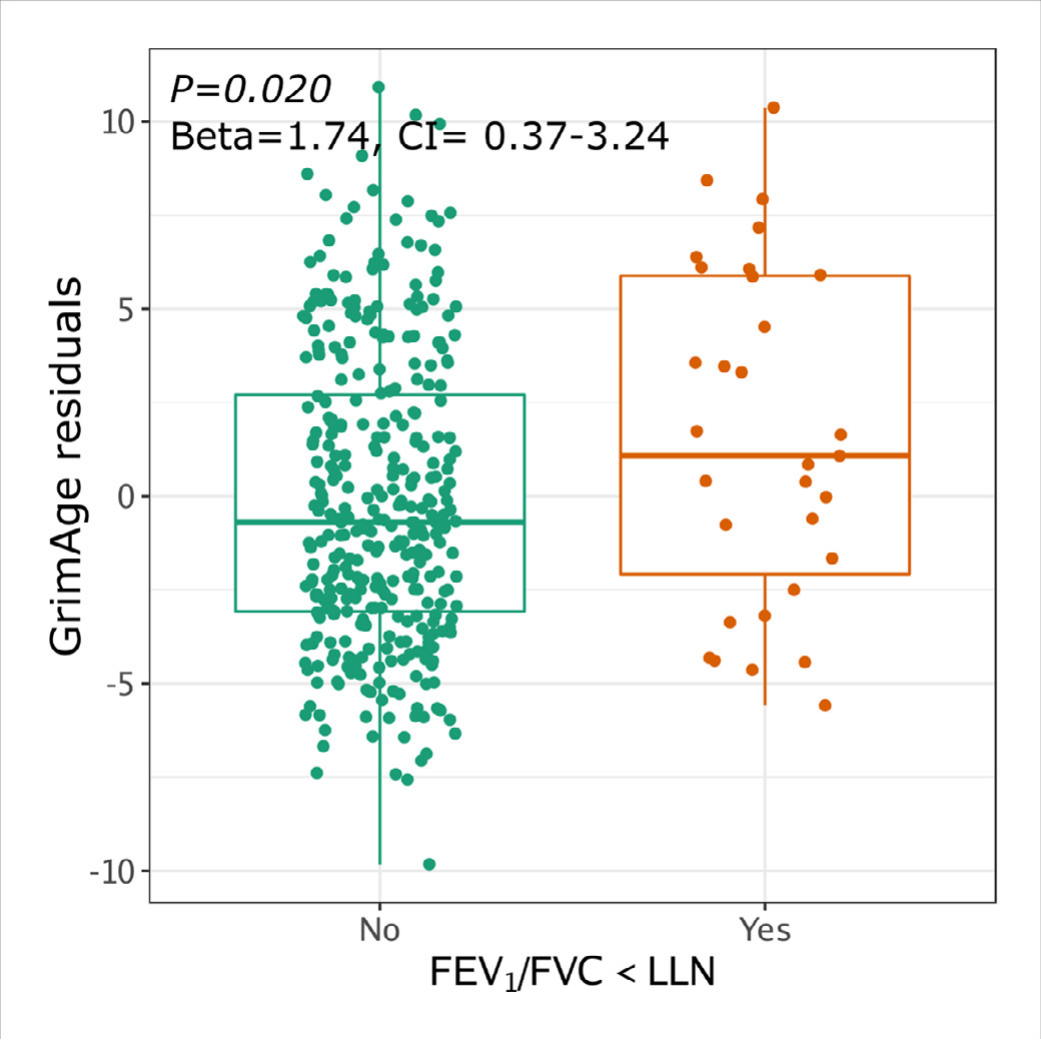
**Blood GrimAge and airflow obstruction in PLWH**. GrimAge residual association with airflow obstruction by the lower limit of normal (LLN) (Airflow obstruction *n*=31, No airfow obstruction *n*=347. P-values, Beta and CI corresponds to the univariate linear regression *p*-value for GrimAge residual (DNAmGrimAge ∼ age + sex + bmi) and airflow obstruction.

In blood, DNAmGrimAge was highly correlated with chronological age (r=0.86, P<0.001) (Supplementary Figure 5). Airflow obstruction by either definition was also associated with greater GrimAge residuals (FEV_1_/FVC<LLN, Beta=1.74, 95%CI=0.37-3.24, *P=*0.019 and FEV_1_/FVC<0.70 Beta=1.80, 95%CI=0.37-3.24, *P=*0.014) (Figure 2, results shown for FEV_1_/FVC<LLN). Lower FEV_1_% predicted (*r*=-0.13, *P=*0.012) and FEV_1_/FVC ratio (*r*=-0.18, *P*<0.001) were associated with increased GrimAge residuals. Furthermore, GrimAge residuals at baseline were statistically significantly associated with faster FEV_1_ decline (*r*=-0.13, *P=*0.012). After adjusting for smoking status, airflow obstruction under the FEV_1_/FVC<LLN definition (Beta=1.13, 95%CI=0.06-2.19, *P=*0.040), FEV_1_% predicted (*r*=-0.17, *P=*0.001) and FEV_1_/FVC ratio (*r*=-0.16, *P=*0.001) remained statistically significant (Supplementary Table 4).

We investigated the ability of blood GrimAge residuals to identify airflow obstruction in PLWH. For airflow obstruction, defined as FEV_1_/FVC<LLN, the AUC based on the GrimAge residuals was modest (AUC=0.61, 95% CI=0.50-0.73); the AUC increased to 0.68 (95% CI=0.58-0.78) with the addition of age, sex, race, BMI, and smoking status. For the airflow obstruction criteria FEV_1_/FVC<0.70, the AUC based on GrimAge residuals alone was 0.62 (95% CI=0.51-0.73), which increased to 0.75 (95% CI 0.65-0.85) using the full model. Supplementary Table 5 shows that GrimAge residuals have a consistent performance over time for the prediction of airflow obstruction. In accordance with these observations, GrimAge residual at baseline was associated with decreased FEV_1_% predicted and FEV_1_/FVC ratio at different time points as the study progressed. For instance, Supplementary Table 6 shows that FEV_1_/FVC at each yearly visit after baseline had a statistically significant correlation with GrimAge residuals at baseline. The AUC for predicting fast FEV1 decline (>40mL/year), though, was modest at 0.60 (95% CI=0.51-0.67) for GrimAge residuals alone. After including age, sex, race, BMI, smoking status, and baseline FEV_1_, the AUC improved to 0.69 (95% CI=0.62-0.77).

**Figure 3:**
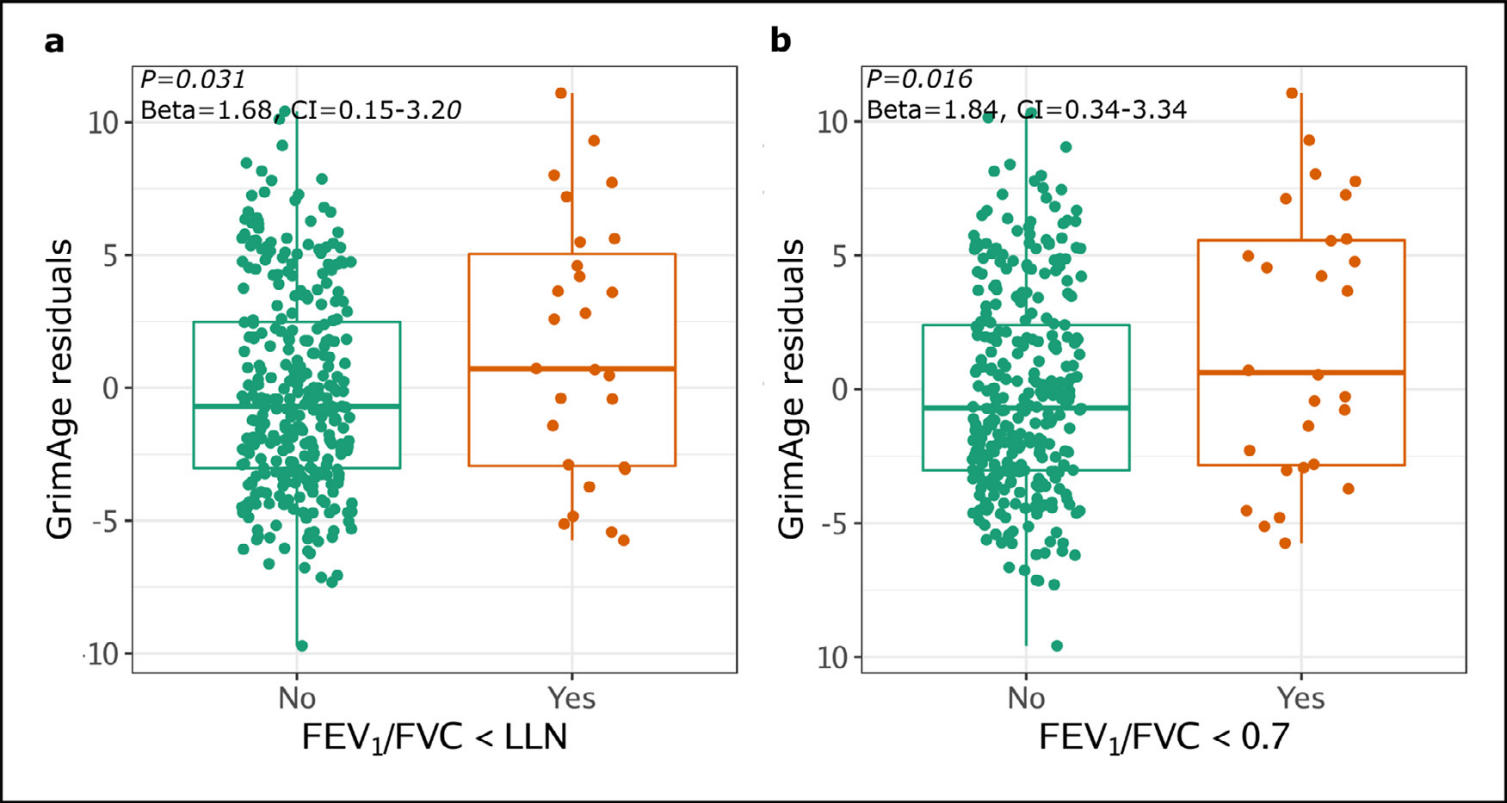
**GrimAge residuals at baseline is associated with subsequent airflow obstruction status**. PLWH who had normal lung function at baseline but who subsequently developed airflow obstruction as defined by an FEV_1_/FVC<LLN (a) or FEV_1_/FVC<0.70 (b) had greater GrimAge residuals at the beginning of the study (*n*=378).

PLWH who had normal lung function at baseline but who developed airflow obstruction in the subsequent years of the study (Years 1 to 6) had statistically significantly higher GrimAge residuals at baseline compared to PLWH who continued to have normal lung function throughout the study (FEV_1_/FVC<LLN Beta=1.68, 95%CI=0.15-3.20, *P=*0.031; FEV_1_/FVC<0.7 Beta=1.84, 95%CI=0.34-3.34, *P=*0.016) (Figure 3). The effect remained statistically significant for FEV_1_/FVC<0.7 after adjusting for smoking (Beta=1.22, 95%CI=0.11-2.34, *P=*0.031), but not for FEV_1_/FVC<LLN (Beta=1.00, 95%CI=-0.13-2.14, *P=*0.084). Supplementary Table 7 shows that the blood GrimAge residuals AUC was still predictive across the six years for both airflow obstruction criteria, although the prediction characteristics were modest at best. For instance, for PLWH whose airways became obstructed at years 4 and 5 (by FEV_1_/FVC<0.7), the AUC based on blood GrimAge residual alone was 0.62 (95%CI 0.39-0.85) and 0.67 (95%CI 0.45-0.89), respectively. When GrimAge residual, age, sex, race, BMI, and smoking status were used in the prediction, the AUC was 0.86 (95%CI 0.70-0.95) for year 4 and 0.97 (95%CI 0.91-1) for year 5. Nevertheless, these results were limited by the sample size and should be interpreted with caution.

We next explored HIV-related factors and comorbidities in the START cohort. There was no association between GrimAge residuals and CD4 cell count, CD4 nadir, CD4/CD8 ratio, Hepatitis C infection and viral load. We did find, however, that the presence of hypertension was associated with higher GrimAge residuals (*P=*0.010).

## Discussion

This study shows that GrimAge of the blood and airway epithelium of PLWH corresponds to their lung health status. The key findings highlight the relationship between advanced GrimAge and airways disease: 1) PLWH with COPD display greater airway epithelial GrimAge residuals compared to PLWH without COPD; 2) lung function in PLWH is statistically significantly inversely correlated with peripheral blood GrimAge residuals; 3) as a biomarker of lung disease, DNAmGrimAge has the ability to translate from airway to blood compartments, suggesting a global epigenetic phenomenon in PLWH; and 4) DNAmGrimAge at baseline is associated with lung function decline over time, thus indicating that changes in blood GrimAge residuals may reflect early lung damage, even prior to the development of airflow obstruction. Although modest in performance for prediction overall, GrimAge residuals at baseline were statistically significantly associated with developing airflow obstruction over the next 6 years. This opens new possibilities that DNAmGrimAge could be used as a complementary prognostic tool to risk stratify PLWH who may go on to develop COPD.

The ability of DNAmGrimAge to reflect lung health status was preserved across two very different HIV cohorts. In the first, DNAmGrimAge was used to show that the epigenetic age acceleration (as defined by Lu and colleagues^16^) in the airway that occurs with COPD persists despite ART and suppressed viral loads. This suggests that while ART can prevent further damage to the immune system by blockading HIV cellular hijacking, ART itself does not reverse airway epigenetic changes completely in PLWH. In the second cohort, we observed that increases in peripheral blood GrimAge residuals for those PLWH with airflow obstruction can occur early on in HIV infection, prior to the reduction in CD4 counts and the development of opportunistic infections and AIDS-related complications. Moreover, these changes can be observed prior to the initiation of ART. These results support our previous observations in a cohort of people using injection drugs that DNA methylation changes can occur very rapidly in HIV soon after HIV seroconversion.^13^ Altogether, these findings suggest that epigenetic disruptions related to airflow obstruction can occur early in HIV, may be independent of ART exposure, and can persist even with viral suppression and immune reconstitution.

As DNAmGrimAge was derived using seven inflammatory plasma proteins that are associated with mortality, it may be better equipped to capture systemic inflammation in COPD than previous iterations of the epigenetic clock.^10^^,^^15^ Systemic inflammation is considered a key feature of COPD,^37^^,^^38^ with one proposed mechanism being the translocation of inflammatory mediators across the lung-blood barrier.^39^, ^40^, ^41^ Cigarette smoking, the main risk factor for COPD, may increase the permeability of pulmonary vessels,^39^^,^^42^ thus contributing to the spillover of inflammatory proteins from the lung to the systemic compartment. Proteins included in the derivation of DNAmGrimAge such as tissue inhibitor metalloproteinases 1 (TIMP-1) have previously demonstrated significant increases in both the sputum^43^ and blood^44^ of individuals with COPD compared to control individuals, perhaps one reason why DNAmGrimAge performs well in both airway and blood compartments in relation to COPD. Other proteins on which DNAmGrimAge was derived, such as Cystatin C and growth differentiation factor‐15, have been associated with COPD^45^ and COPD exacerbations^46^ in blood. For PLWH specifically, HIV infection likely represents an additional pro-inflammatory factor driving these lung effects. A previous analysis of START pulmonary sub-study participants, for instance, identified elevated plasma inflammatory biomarkers such as D-dimer and interleukin-6 in PLWH with airflow obstruction.^47^

Our study had several limitations. First, while most participants in the bronchoscopy cohort were on ART, the START cohort was not treated with ART at baseline^22^ and thus the results of the peripheral blood analysis may not be generalizable to PLWH on ART with suppressed viral loads and reduced chronic inflammation. These results may also not be generalizable to PLWH who have had longer durations of HIV infection. Second, we did not explore DNAmGrimAge longitudinally in either cohort so whether DNAmGrimAge can fluctuate over time in response to infections, exacerbations, or additional inhalational exposures remains unknown. Third, neither of the cohorts used for this study were profiled for both tissues, thus the correlation of airway and blood DNAmGrimAge within an individual is uncertain. Fourth, co-infections, comorbidities and unaccounted confounders may represent additional factors contributing to GrimAge residuals and their association with lung function. Given that the START cohort was relatively healthy with only recent diagnoses of HIV, there were few opportunistic infections or comorbidities, however, to test this hypothesis. Fifth, while the START cohort represented PLWH from multiple nations, our bronchoscopy cohort was limited to a single center and further validation of our airway epithelial findings in PLWH from other regions of the world is warranted. Lastly, there is still no accepted minimal clinically important difference in epigenetic age, therefore we do not know whether the magnitude of difference in GrimAge has prospective clinical significance. Future work should determine whether GrimAge residuals are associated not only with COPD and lung function decline in PLWH, but also important clinical outcomes such as hospitalizations and death.

Despite these limitations, our findings suggest that a global increases in airway and blood GrimAge are associated with lung function and COPD status in PLWH. Our results indicate that epigenetic regulation of ageing and mortality-associated mechanisms may contribute to the unique pathophysiology of COPD in PLWH. Together, our findings suggest that DNAmGrimAge could potentially help to identify PLWH at risk of COPD.

## Contributors

Ana I Hernandez Cordero, Steve Horvath, Tawimas Shaipanich, Michael S Kobor, Silvia Guilemi, Marianne Harris, Wan Lam, Stephen Lam, Ma'en Obeidat, Julio Montaner, S.F. Paul Man, Ken Kunisaki, Don D Sin and Janice M Leung contributed to the study design and conception. Ana I Hernandez Cordero, Julia Yang, Tawimas Shaipanich, Julie MacIsaac, David Lin, Lisa McEwen, Michael S Kobor, Silvia Guillemi, Marianne Harris, Richard M Novak, Fleur Hudson, Hartwig Klinker, Nila Dharan, Julio Montaner, S.F. Paul Man, Ken Kunisaki, Don D Sin, Janice M Leung contributed to the data collection. Data analyses were performed by Ana I Hernandez Cordero, Chen Xi Yang, Julia Yang, Xuan Li, Steve Horvath, Julie MacIsaac, David Lin, Lisa McEwen, Michael S Kobor, Ma'en Obeidat, Ken Kunisaki, Janice M Leung. Ana I Hernandez Cordero, Chen Xi Yang, and Janice M Leung wrote the first manuscript draft and verified the underlying data. All authors have revised, edited, read, and approved the final version of the manuscript.

## Data sharing statement

All data supporting this study are included in this manuscript and in the supplementary data. The full methylation data used for the START blood samples will be deposited into a public repository in 2022 upon conclusion of the START study. Airway epithelium DNA methylation data are available in the GEO database (GSE178807). GrimAge is available from the non-profit Epigenetic Clock Development Foundation (https://clockfoundation.org) to qualified academic investigators for replication and validation studies. Metadata are available upon reasonable request directed to Dr. Janice Leung at Janice.Leung@hli.ubc.ca.

## Declaration of interests

SH reports consulting fees from the non-profit organization, Epigenetic Clock Development Foundation, and royalty income from patents surrounding epigenetic clocks. HK reports consulting fees and payments from AbbVie, Janssen, MSD, ViiV, Gilead, and Intercept, outside of the submitted work. KK reports consulting fees and payments from Allergan and Nuvaira, outside of the submitted work. DS reports payments from AstraZeneca, GSK, and Boehringer Ingelheim, outside of the submitted work. MH reports consulting fees and payments from Gilead, Merck, and ViiV, outside of the submitted work. RMN reports payments from ViiV, Gilead, and Theratechnologies, outside of the submitted work. J Montaner reports grants from Gilead, Merck, ViiV, the Public Health Agency of Canada, and the BC Ministry of Health, outside of the submitted work. JML reports a grant from AstraZeneca, outside of the submitted work. The remaining authors report no competing interests.

## Appendix Supplementary materials

Image, application 1

Image, application 2

Supplementary Figure 1. Diagram represents the observational cohort study design used. Our analyses are based on two study cohorts, cohort 1: St. Paul's Hospital HIV Bronchoscopy study, and cohort 2: The Strategic Timing of Antiretroviral Treatment (START) study. People living with HIV (PLWH) were selected from cohort 1 and characterized as COPD positive or negative. From cohort 2, all PLWH that were part of the Pulmonary and Genomic sub-studies were selected for downstream analyses. Participants from cohort 1 provided cross-sectional bronchoscopic specimens and pulmonary function testing. Participants from cohort 2 provided baseline blood samples and pulmonary function testing (Year 1 to Year 6). Cohort 1 and 2 were profiled for airway and blood DNA methylation, respectively; based on the methylation profiles, epigenetic age (DNAmGrimAge) was calculated for the participants in each cohort. Image, application 3

Supplementary Figure 2. Epigenetic age interpretation. The diagram illustrates how epigenetic age (y-axis) can differ from chronological age (x-axis). The difference or residuals distance between an individual's epigenetic age and chronological age indicates either younger or older epigenetic age compared to chronological age. Image, image 4

Supplementary Figure 3. The correlation between airway epithelium DNAmGrimAge and chronological age (*n*=34). The correlation coefficient is shown in the top left corner of the figure next to the corresponding p-value obtained from a univariate linear model (DNAmGrimAge ∼ Chronological age). Image, image 5

Supplementary Figure 4. Receiver operating characteristic (ROC) curves and area under the curve (AUC) for the prediction of COPD in the airway epithelium of PLWH. (a) ROC curve for GrimAge residuals only. (b) ROC curve for chronological age, sex, BMI, and smoking status. (c) ROC curve for the full model of GrimAge residuals, chronological age, sex, BMI, and smoking status. Image, image 6

Supplementary Figure 5. Correlation between Blood DNAmGrimAge and chronological age (*n*=378). The correlation coefficient is shown at the top left corner of the figure next to the corresponding p-value obtained from a univariate linear model (DNAmGrimAge ∼ Chronological age). Image, image 7

Image, image 8
