# Supplementary material for: The relationship between the epigenetic aging biomarker “grimage” and lung function in both the airway and blood of people living with HIV: An observational cohort study
Source: eBioMedicine. 2022 Aug 6;83:104206. doi: 10.1016/j.ebiom.2022.104206 (PMC9379521; doi:10.1016/j.ebiom.2022.104206)
Supplement: Supplementary file 1 [file mmc1.docx]

**SUPPLEMENTARY TABLES**

**Supplementary Table 1. Variables included in the analyses.**

| **Response variable** | **Independent variables** | **Analysis** | **Cohort** |
| --- | --- | --- | --- |
| Airway epithelium | | | |
| GrimAge residual (**DNAmGrimAge ~ Age + Sex + BMI**) | COPD | Linear regression | St. Paul’s Hospital HIV Bronchoscopy study cohort |
| GrimAge residual | FEV_1_%pre | Linear regression |  |
| GrimAge residual | FEV_1_/FVC | Linear regression |  |
| COPD | GrimAge residual (**DNAmGrimAge ~ Age + Sex +BMI**) | Operating characteristic curve /Area under the curve |  |
| COPD | Chronological Age + Sex + BMI + Smoking status | Operating characteristic curve /Area under the curve |  |
| COPD | GrimAge residual (**DNAmGrimAge ~ Age + Sex +BMI**) + Chronological Age + Sex + BMI + Smoking status | Operating characteristic curve /Area under the curve |  |
| COPD | GrimAge residual (**DNAmGrimAge ~ Age + Sex +BMI + Smoking status)** | Operating characteristic curve /Area under the curve |  |
| Blood | | | |
| GrimAge residual  (**DNAmGrimAge ~ Age + Sex + Race + BMI + Cell proportions**) | Airflow obstruction (FEV_1_/FVC<LLN) | Linear regression | The Strategic Timing of Antiretroviral Treatment (START) study cohort |
| GrimAge residual | Airflow obstruction (FEV_1_/FVC<0.70) | Linear regression |  |
| GrimAge residual | FEV_1_%pre | Linear regression |  |
| GrimAge residual | FEV_1_/FVC | Linear regression |  |
| FEV_1_ decline | GrimAge residual | Operating characteristic curve /Area under the curve |  |
| Airflow obstruction (FEV_1_/FVC<LLN) | GrimAge residual | Operating characteristic curve /Area under the curve |  |
| Airflow obstruction  (FEV_1_/FVC<0.70) | GrimAge residual | Operating characteristic curve /Area under the curve |  |

**Supplementary Table 2. START cohort demographics based on the FEV_1_/FVC<0.70 criteria.**

| START cohort (N=378) | | |
| --- | --- | --- |
|  | Airflow obstruction (FEV1/FVC<0.70) | No airflow obstruction  (FEV1/FVC≥0.70) |
| N | 32 | 346 |
| Age (median, IQR) | 42  (35-50) | 36  (31-45) |
| Females, % | 9% | 12% |
| Smoking status  Current, %  Former, %  Never, % | 66%  9%  25% | 44%  19%  37% |
| Pack-Year History | 11  (0-28) | 2  (0-9) |
| Race  Black, %  Asian, %  Caucasian, %  Hispanic, %  Other, % | 13%  0%  78%  9%  0% | 18%  1%  61%  17%  2% |
| BMI (kg/m^2^) | 22.98  (21.69-25.62) | 24.57  (22.49-27.53) |
| FEV_1_, ml | 3185  (2525-3872) | 3830  (3350-4312) |
| FEV_1_, % predicted | 81.68%  (68.29-90.41) | 96.42%  (88.88-103.99) |
| FVC, ml | 4895  (4035-5915) | 4685  (4120-5338) |
| FVC, % predicted | 97.69%  (87.91-109.76) | 95.98%  (87.94-103.59) |
| FEV_1_/FVC ratio | 0.67  (0.65-0.69) | 0.81  (0.78-0.86) |
| Hepatitis C | 3% | 4% |
| Hypertension | 3% | 10% |
| CD4 T cells/mm^3^ | 666.0  (588.6-752.9) | 636.8  (582.8-736.9) |
| HIV RNA viral load, copies/mm^3^ | 23050  (5096-72000) | 16422  (4450-56100) |

Spirometry corresponds to pre-bronchodilator measurements. BMI: body mass index. SD: standard deviation. Median and interquartile range are shown for non-normally distributed variables. Race was based on the participant’s self-assessment.

**Supplementary Table 3. GrimAge residuals association with COPD and lung function in PLWH adjusted for inhaled corticosteroid use.**

| **Tissue** | **Trait** | **r** | **Beta** | **CI** | **P*** |
| --- | --- | --- | --- | --- | --- |
| Airway | COPD | *-* | *3.20* | *1.14-5.27* | *0.004* |
|  | FEV_1_ | *-0.35* | *-0.05* | *-* | *0.054* |
|  | FEV_1_/FVC | *-0.44* | *-0.12* | *-* | *0.013* |

*The P-value corresponds to the relationship between GrimAge residuals (DNAmGrimAge ~ age + sex + BMI + inhaled corticosteroid use) and each trait (COPD, FEV_1_, FEV_1_/FVC). GrimAge tests correspond to the univariate linear regression model between GrimAge residuals ~ lung function trait (n=31).

**Supplementary Table 4. GrimAge residuals association with lung function traits in PLWH adjusted for smoking status.**

| **Tissue** | **Trait** | **r** | **Beta** | **95% CI** | **P*** |
| --- | --- | --- | --- | --- | --- |
| Blood | FEV_1_/FVC<0.70 | *-* | *0.91* | *-0.141-1.969* | *0.084* |
|  | FEV_1_/FVC<LLN | *-* | *1.13* | *0.057- 2.194* | *0.040* |
|  | FEV_1_%predicted | *-0.17* | *-0.04* | *-* | *<0.001* |
|  | FEV_1_/FVC | *-0.16* | *-7.01* | *-* | *0.002* |
|  | FEV_1_ change (mL/year) | *-0.02* | *-0.0005* | *-* | *0.741* |

*The P-value corresponds to the relationship between DNAmGrimAge and each lung function trait in the START study cohort. DNAmGrimAge was adjusted for age, sex, race, BMI, and smoking status (current/former/never) and blood cell proportions. GrimAge tests corresponded to univariate linear regression model between GrimAge residuals ~ lung function trait (n=378).

**Supplementary Table 5.** **GrimAge residuals as a predictor of airflow obstruction.**

| Year | FEV_1_/FVC Criteria | N  (AO) | N  (no AO) | AUC (95%CI)  GrimAge  residuals | AUC (95%CI)  Demographics  Only* | AUC (95%CI)  Full model* |
| --- | --- | --- | --- | --- | --- | --- |
| Y0 | <0.70 | 32 | 346 | 0.62 (0.51-0.73) | 0.74 (0.64-0.84) | 0.75 (0.65-0.85) |
|  | <LLN | 31 | 347 | 0.61 (0.50-0.73) | 0.66 (0.56-0.76) | 0.68 (0.58-0.78) |
| Y1 | <0.70 | 31 | 327 | 0.62 (0.51-0.73) | 0.77 (0.68-0.86) | 0.78 (0.69-0.87) |
|  | <LLN | 30 | 329 | 0.62 (0.51-0.73) | 0.64 (0.55-0.75) | 0.68 (0.58-0.77) |
| Y2 | <0.70 | 27 | 323 | 0.52 (0.40-0.64) | 0.72 (0.62-0.83) | 0.74 (0.62-0.83) |
|  | <LLN | 28 | 322 | 0.56 (0.44-0.68) | 0.71 (0.61-0.82) | 0.71 (0.61-0.82) |
| Y3 | <0.70 | 24 | 305 | 0.69 (0.59-0.80) | 0.80 (0.69-0.92) | 0.80 (0.70-0.91) |
|  | <LLN | 21 | 308 | 0.64 (0.51-0.77) | 0.77 (0.65-0.89) | 0.77 (0.65-0.89) |
| Y4 | <0.70 | 22 | 228 | 0.63 (0.49-0.78) | 0.76 (0.66-0.87) | 0.80 (0.71-0.90) |
|  | <LLN | 21 | 229 | 0.61 (0.46-0.75) | 0.72 (0.60-0.83) | 0.77 (0.68-0.87) |
| Y5 | <0.70 | 18 | 127 | 0.64 (0.48-0.79) | 0.86 (0.76-0.96) | 0.87 (0.77-0.97) |
|  | <LLN | 15 | 130 | 0.65 (0.47-0.83) | 0.84 (0.73-0.95) | 0.85 (0.75-0.96) |
| Y6* | <0.70 | 6 | 45 | 0.82 (0.67-0.96) | 0.91 (0.83-1) | 0.91 (0.79-1) |
|  | <LLN | 5 | 46 | 0.83 (0.67-0.99) | 0.90 (0.81-1) | 0.90 (0.77-1) |

*At year 6, the sample size was not sufficient for race adjustment. AO=airflow obstruction. GrimAge residuals used for the calculation of AUC was defined as the residual obtained from the regression of DNAmGrimAge on chronological age, sex, and BMI.

*Demographics only model included age, sex, race, BMI, and smoking status, while the full model included GrimAge residuals, age, sex, race, BMI, and smoking status.

**Supplementary Table 6. Blood GrimAge residuals association with lung function at different timepoints during the START study.**

| Year | Variable | r | Beta | P-value |
| --- | --- | --- | --- | --- |
| Y1 | **FEV_1_%** | -0.16 | -0.05 | 0.002 |
| Y2 |  | -0.17 | -0.05 | 0.002 |
| Y3 |  | -0.11 | -0.03 | 0.044 |
| Y4 |  | -0.18 | -0.05 | 0.005 |
| Y5 |  | -0.24 | -0.07 | 0.004 |
| Y6 |  | -0.21 | -0.07 | 0.135 |
| Y1 | **FEV_1_/FVC** | -0.20 | -9.96 | <0.001 |
| Y2 |  | -0.20 | -9.16 | <0.001 |
| Y3 |  | -0.17 | -8.86 | 0.001 |
| Y4 |  | -0.24 | -11.00 | <0.001 |
| Y5 |  | -0.24 | -10.59 | 0.004 |
| Y6 |  | -0.35 | -16.48 | 0.013 |
|  |  |  |  |  |

This table shows that at baseline (Year 0), GrimAge residuals (DNAmGrimAge ~ Age + Sex + Race + BMI + cell proportions) have a statistically significant association with lung function (FEV_1_ and FEV_1_/FVC) over time based on six yearly visits (Year 1, 2, 3, 4, 5 and 6). Betas and P-values correspond to the univariate linear regression between GrimAge and lung function trait.

**Supplementary Table 7.** **GrimAge residuals as a predictor of airflow obstruction in patients with normal lung function at baseline (FEV1/FVC >LLN or >0.70) but who developed subsequent airflow obstruction.**

| Year | Criteria | N (AO) | N (no AO) | AUC (95%CI)  GrimAge residuals | AUC (95%CI)  Demographics only* | AUC (95%CI)  Full model* |
| --- | --- | --- | --- | --- | --- | --- |
| Y1 | FEV_1_/FVC<0.70 | 12 | 317 | 0.57 (0.38-0.76) | 0.83 (0.72-0.94) | 0.84 (0.74-0.96) |
|  | FEV_1_/FVC<LLN | 13 | 317 | 0.66 (0.49-0.82) | 0.71 (0.58-0.84) | 0.75 (0.63-0.88) |
| Y2 | FEV_1_/FVC<0.70 | 9 | 315 | 0.49 (0.25-0.73) | 0.75 (-.59-0.90) | 0.75 (0.60-0.90) |
|  | FEV_1_/FVC<LLN | 12 | 311 | 0.53 (0.33-0.73) | 0.78 (0.66-0.90) | 0.78 (0.68-0.89) |
| Y3 | FEV_1_/FVC<0.70 | 3 | 296 | 0.69 (0.48-0.90) | 0.97 (0.93-1) | 0.97 (0.94-1) |
|  | FEV_1_/FVC<LLN | 3 | 296 | 0.58 (0.19-0.97) | 0.92 (0.81-1) | 0.97 (0.94-1) |
| Y4 | FEV_1_/FVC<0.70 | 9 | 220 | 0.62 (0.39-0.85) | 0.79 (0.67-0.91) | 0.86 (0.70-0.95) |
|  | FEV_1_/FVC<LLN | 7 | 221 | 0.46 (0.17-0.75) | 0.76 (0.63-0.89) | 0.81 (0.71-0.91) |
| Y5 | FEV_1_/FVC<0.70 | 7 | 123 | 0.67 (0.45-0.89) | 0.91 (0.85-0.99) | 0.97 (0.91-1) |
|  | FEV_1_/FVC<LLN | 5 | 125 | 0.59 (0.19-0.99) | 0.90 (0.84-0.97) | 0.93 (0.89-0.98) |
| Y6* | FEV_1_/FVC<0.70 | 4 | 42 | 0.78 (0.60-0.96) | 0.91 (0.82-0.99) | 0.91 (0.77-1) |
|  | FEV_1_/FVC<LLN | 3 | 42 | 0.79 (0.56-1) | 0.92 (0.84-1) | 0.89 (0.73-1) |

*At year 6, the sample size was not sufficient for race adjustment. AO=airflow obstruction. GrimAge residuals used for the calculation of AUC was defined as the residual obtained from the regression of DNAmGrimAge on chronological age, sex, and BMI.

*Demographics only model included age, sex, race, BMI, and smoking status, while the full model included GrimAge residuals, age, sex, race, BMI, and smoking status.
