# Supplementary figures and images for: The relationship between the epigenetic aging biomarker “grimage” and lung function in both the airway and blood of people living with HIV: An observational cohort study
Source: eBioMedicine. 2022 Aug 6;83:104206. doi: 10.1016/j.ebiom.2022.104206 (PMC9379521; doi:10.1016/j.ebiom.2022.104206)

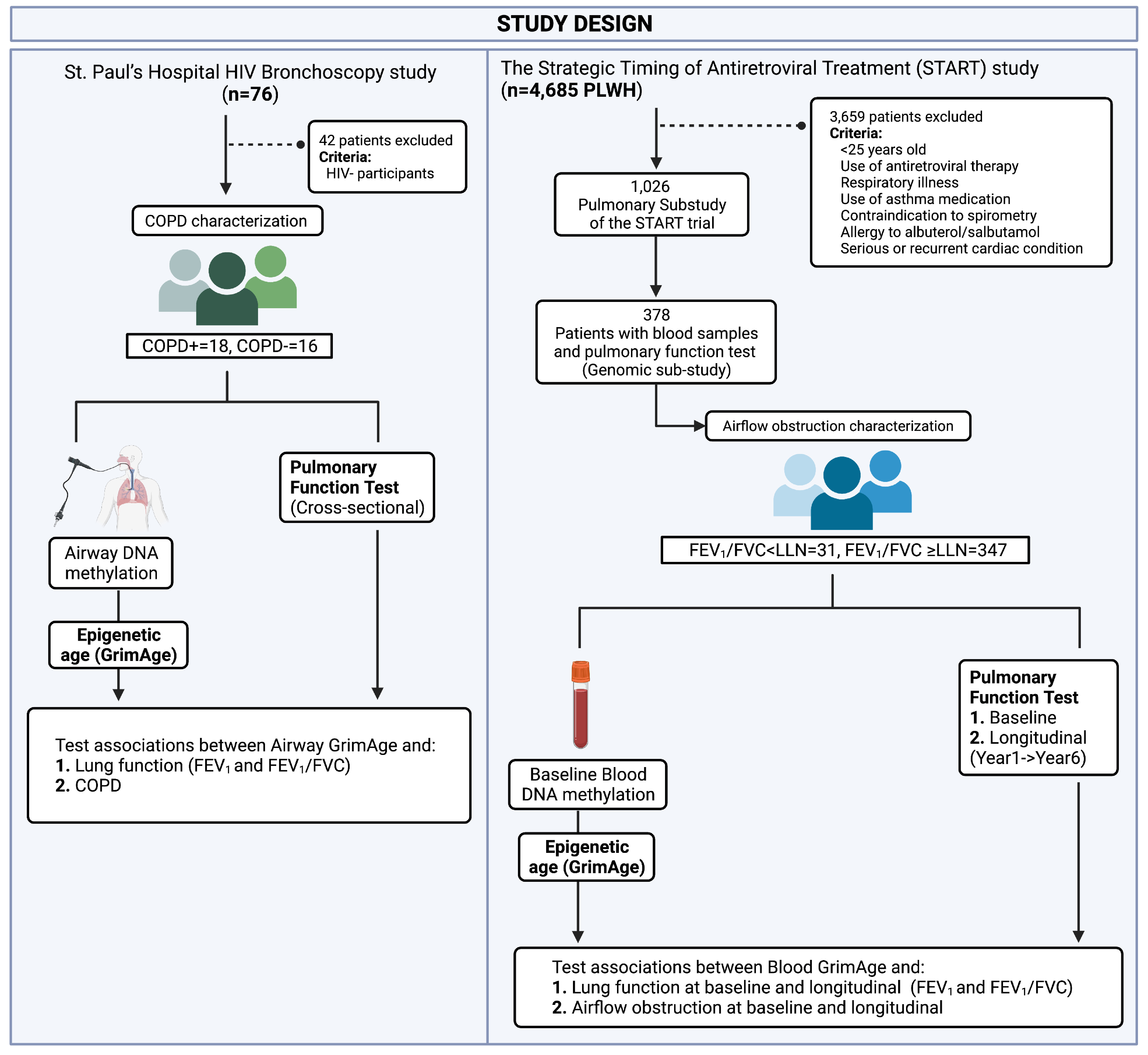

Supplement: Supplementary file 4 — Supplementary Figure 2. Epigenetic age interpretation. The diagram illustrates how epigenetic age (y-axis) can differ from chronological age (x-axis). The difference or residuals distance between an individual's epigenetic age and chronological age indicates either younger or older epigenetic age compared to chronological age. [file mmc4.jpg]

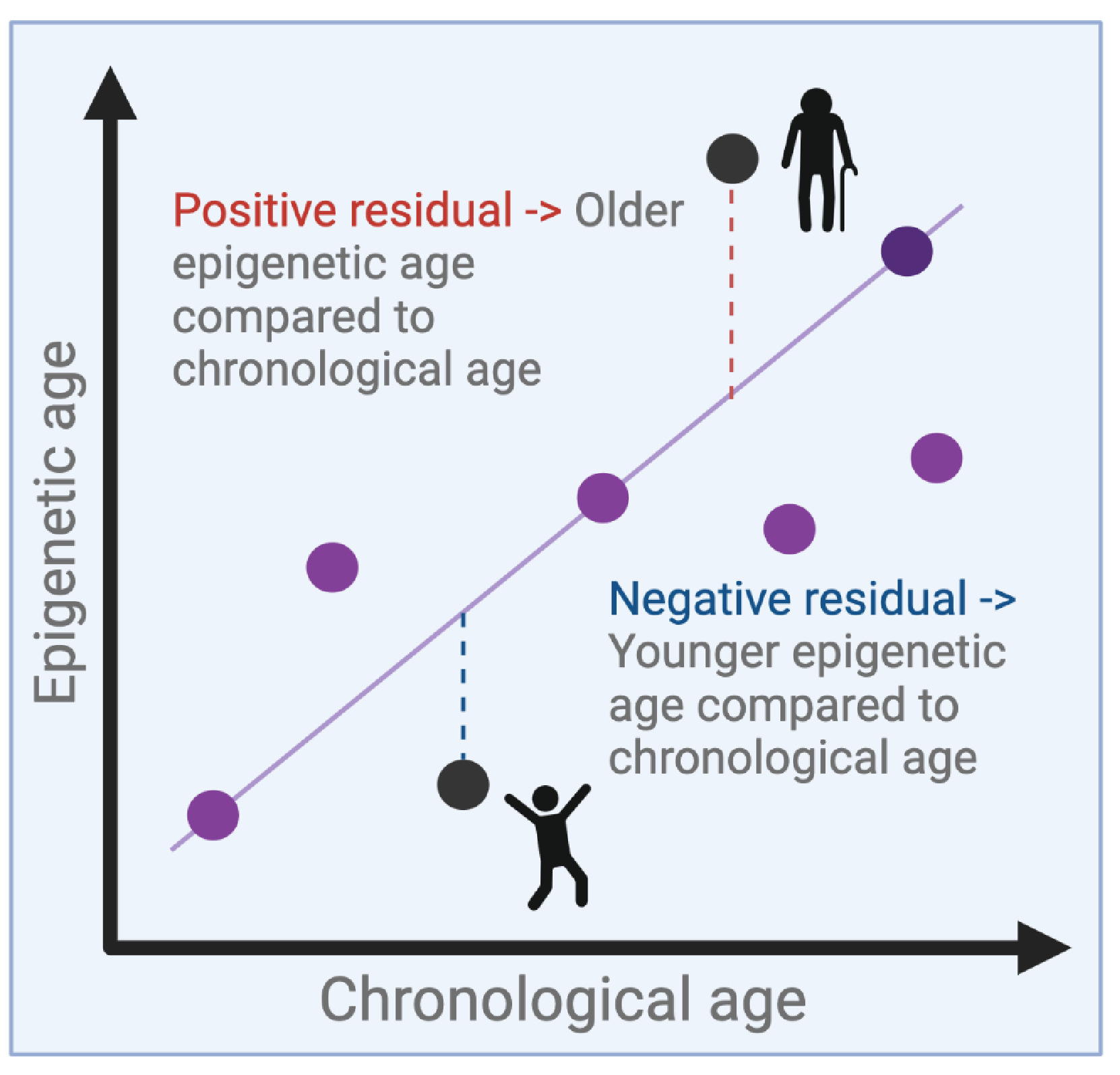

Supplement: Supplementary file 5 — Supplementary Figure 3. The correlation between airway epithelium DNAmGrimAge and chronological age (n=34). The correlation coefficient is shown in the top left corner of the figure next to the corresponding p-value obtained from a univariate linear model (DNAmGrimAge ∼ Chronological age). [file mmc5.jpg]

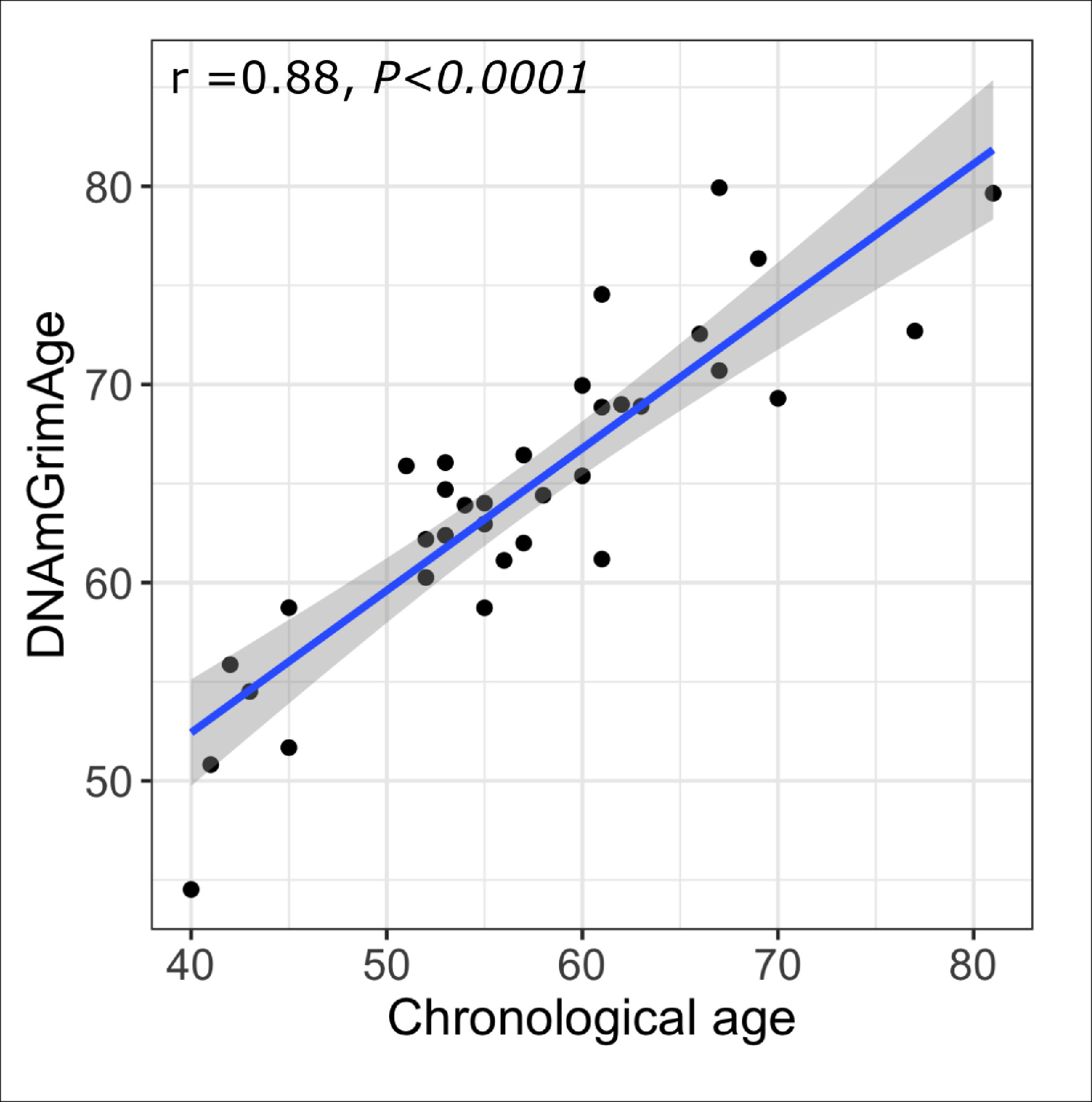

Supplement: Supplementary file 6 — Supplementary Figure 4. Receiver operating characteristic (ROC) curves and area under the curve (AUC) for the prediction of COPD in the airway epithelium of PLWH. (a) ROC curve for GrimAge residuals only. (b) ROC curve for chronological age, sex, BMI, and smoking status. (c) ROC curve for the full model of GrimAge residuals, chronological age, sex, BMI, and smoking status. [file mmc6.jpg]

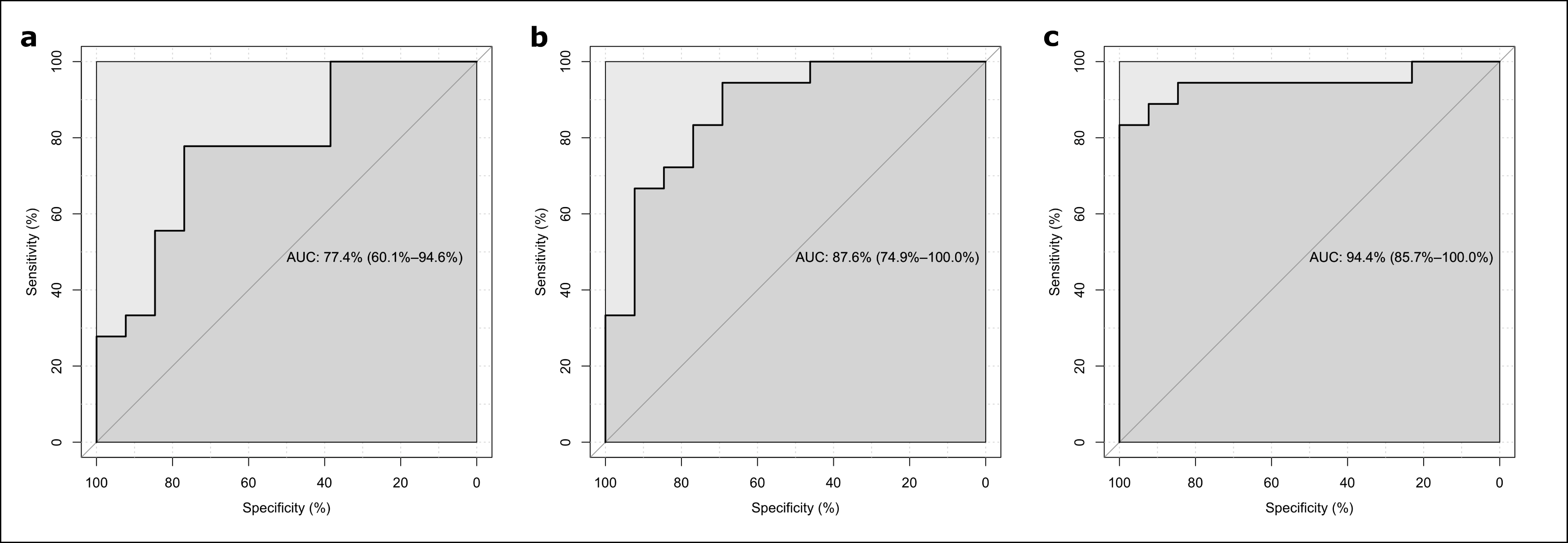

Supplement: Supplementary file 7 — Supplementary Figure 5. Correlation between Blood DNAmGrimAge and chronological age (n=378). The correlation coefficient is shown at the top left corner of the figure next to the corresponding p-value obtained from a univariate linear model (DNAmGrimAge ∼ Chronological age). [file mmc7.jpg]

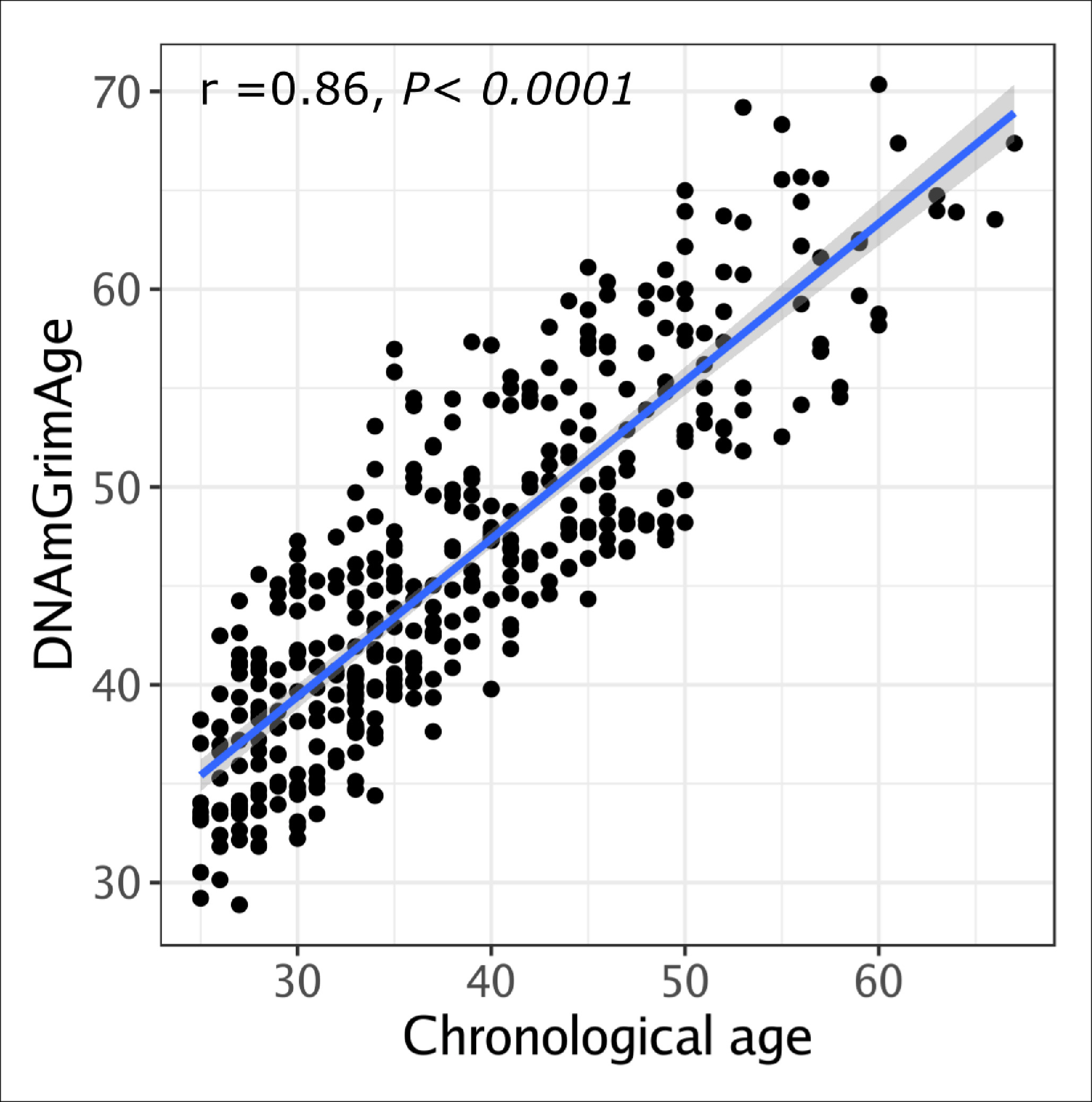

Supplement: Supplementary file 8 [file mmc8.jpg]
